# Thermodynamic and Spectroscopic Investigation of Interactions between Reactive Red 223 and Reactive Orange 122 Anionic Dyes and Cetyltrimethyl Ammonium Bromide (CTAB) Cationic Surfactant in Aqueous Solution

**DOI:** 10.1155/2014/540975

**Published:** 2014-08-28

**Authors:** Muhammad Irfan, Muhammad Usman, Asim Mansha, Nasir Rasool, Muhammad Ibrahim, Usman Ali Rana, Mohammad Siddiq, Muhammad Zia-Ul-Haq, Hawa Z. E. Jaafar, Salah Ud-Din Khan

**Affiliations:** ^1^Department of Chemistry, Government College University, Faisalabad 38000, Pakistan; ^2^Department of Environmental Sciences, Government College University, Faisalabad 38000, Pakistan; ^3^Deanship of Scientific Research, College of Engineering, King Saud University P.O. Box 800, Riyadh 11421, Saudi Arabia; ^4^Department of Chemistry, Quaid-i-Azam University, Islamabad 45320, Pakistan; ^5^The Patent Office, Karachi 74400, Pakistan; ^6^Department of Crop Science, Faculty of Agriculture, UPM, 43400 Serdang, Selangor, Malaysia

## Abstract

The present study describes the conductometric and spectroscopic study of the interaction of reactive anionic dyes, namely, reactive red 223 and reactive orange 122 with the cationic surfactant cetyltrimethyl ammonium bromide (CTAB). In a systematic investigation, the electrical conductivity data was used to calculate various thermodynamic parameters such as free energy (Δ*G*), enthalpy (Δ*H*), and the entropy (Δ*S*) of solubilization. The trend of change in these thermodynamic quantities indicates toward the entropy driven solubilization process. Moreover, the results from spectroscopic data reveal high degree of solubilization, with strong interactions observed in the cases of both dyes and the CTAB. The spontaneous nature of solubilization and binding was evident from the observed negative values of free energies (Δ*G*_*p*_ and Δ*G*_*b*_).

## 1. Introduction

The study of dye/surfactant interaction is the source of useful information to understand several industrial processes, for example, solubilization processes to remove the organic compounds from aqueous solution and the use of surfactants to assist dying processes in textile industry [1]. The efficient removal of loosely bound dyes from the substrate via solubilization and the adsorption and fixation of dyes on a substrate highly depend on the strength of binding between dyes and the host as well as the extent of dye solubilization in the surfactant containing solution [2]. By making use of their polar as well as nonpolar moieties, the micelles provide heterogeneous media to solubilize organic compounds during dye removal process [3]. In premicellar region, the surfactant monomers interact with the dye molecules to form ion association complexes, while in postmicellar region, the dye molecules likely incorporate into the micelles [4]. In such cases, the hydrophobic effect is the dominant factor to decide the locus of additive within micelle. However, the effects of electrostatic interactions are not also too weak to be ignored [5].

Reactive dyes have reactive functional groups capable of forming covalent bond with substrate. Acidic and basic conditions are important for successful and rapid reaction of dyes with substrate [6]. Reactive dyes are most commonly used in dyeing of cellulose like cotton or flax. They can also be applied on wool and nylon, where, in the latter case, they are applied under weakly acidic conditions [7].

Earlier, we have reported the solubilization of some drugs, that is, chloroquine diphosphate, quinacrine 2HCl, and pefloxacin mesylate [8–10] by micellar solutions. In the present study, we report the detailed investigation of the interaction of reactive dyes, namely, reactive orange 122 (RO122) and reactive red 223 (RR223) with cationic surfactant cetyltrimethyl ammonium bromide (CTAB).

## 2. Parameters Calculated

### 2.1. Calculation of Thermodynamic Parameters

Free energy of solubilization (Δ*G*) was calculated using the following equation;

(1) $$\begin{array}{c} \Delta G=\left(2-\beta \right)RT\ln X_{\text{CMC}}. \end{array}$$

In (1), *β* is the degree of dissociation; *R* is the general gas constant having value ~8.314 Jmol^−1^ K^−1^; *T* is the absolute temperature; *X*_CMC_ is CMC in term of mole fraction. Here, *β* can be calculated from the ratio of the slopes of postmicellar and premicellar regions of conductivity-concentration plot using following equation:

(2) $$\begin{array}{c} \beta =\frac{S_{2}}{S_{1}}. \end{array}$$

In (2), *S*_1_ and *S*_2_ represent the slopes of the straight lines in the premicellar and postmicellar region, respectively.

The value of enthalpy of solubilization can be obtained from the following equation [8–15]:

(3) $$\begin{array}{c} \Delta H=-2.3\left(2-\beta \right)RT^{2}{\left[\frac{\partial \left(\log X_{\text{CMC}}\right)}{\partial T}\right]}_{P}. \end{array}$$

In (3), the factor ∂(log⁡*X*_CMC_)/∂*T* was obtained from the slope of straight line plotted between log⁡(*X*_CMC_) and *T*. The entropy of micellization can be calculated by the following equation:

(4) $$\begin{array}{c} \Delta S=\frac{\Delta H-\Delta G}{T}. \end{array}$$

### 2.2. Calculation of Partition and Binding Parameters

Partitioning of dye molecules between aqueous and micellar media is governed by partition law. Partition coefficient is determined by differential absorbance method reported by Kawamura et al. [12]:

(5) $$\begin{array}{c} \frac{1}{\Delta A}=\frac{1}{K_{c}\Delta A_{\infty }\left(C_{d}+C_{s}^{mo}\right)}+\frac{1}{\Delta A_{\infty }}. \end{array}$$

In (5), *C*_*d*_ is concentration of dye in mol*·*dm^−3^ and *C*_*s*_^*mo*^ represents *C*_*s*_-CMC_*o*_, in the same units. Here, CMC_*o*_ is CMC of CTAB in water and *C*_*s*_ is total surfactant concentration in mol*·*dm^−3^. Δ*A* is differential absorbance, and Δ*A*_*∞*_ represents its value at infinity. *K*_*c*_ is partition constant having value in dm^3^/mol. The dimensionless quantity partition coefficient *K*_*x*_ is obtained as *K*_*x*_ = *K*_*c*_ *n*_*w*_, where *n*_*w*_ is number of moles of water per dm^3^.

The value of standard free energy change for the transfer of additive from aqueous to micellar phase was calculated using the following relation:

(6) $$\begin{array}{c} \Delta G_{p}=-RT\ln K_{x}. \end{array}$$

In (6), *R* is the general gas constant and *T* is absolute temperature. Later, the binding constant was calculated by using the following quantitative approach:

(7) $$\begin{array}{c} \frac{C_{s}C_{d}}{\Delta A}=\frac{C_{s}}{\Delta \varepsilon l}+\frac{1}{K_{b}\Delta \varepsilon l}. \end{array}$$

In (7), *C*_*d*_ represents the concentration of dye, while *C*_*s*_ indicates the surfactant concentration. In addition, Δ*A* represents differential absorbance; Δ*ε* is difference of absorption coefficient; *l* is path length, while *K*_*b*_ stands for the binding constant [12–14].

The value of standard free energy change of binding was calculated using the following relation:

(8) $$\begin{array}{c} \Delta G_{b}=-RT\ln K_{b}. \end{array}$$

## 3. Materials and Method

### 3.1. Materials and Preparation of Solution

Specific conductivities were measured with WTW inoLab Cond 720 (Germany). This instrument can measure the electrical conductivity in the range of 0.01*μ*S to 199.9 mS with an accuracy of ±0.5% ± 2 and temperature control accuracy of ~±0.5 K. The electrode used has cell constant ~0.98 cm^−1^ and was coated with platinum black to avoid the polarization effect. The conductivities were measured over the temperature range ~293 K–323 K with the increment of 10 K. The electrode was calibrated using KCl_(aq)_ over the appropriate concentration range.

All absorption spectra of the sample were measured on Hitachi UV-2800 spectrophotometer in the UV-visible range. The cells used were square cuvettes having 1.0 cm in thickness with the slit width of ~1.0 nm. In simple UV/visible absorption spectra, distilled water was kept at the reference side, while, in the differential UV/visible spectroscopy, dye solution was adopted as reference. In both cases, dye-surfactant-water ternary solution was taken in the sample cell.

## 4. Results and Discussion

### 4.1. Conductometric Studies

**Figure 1:**
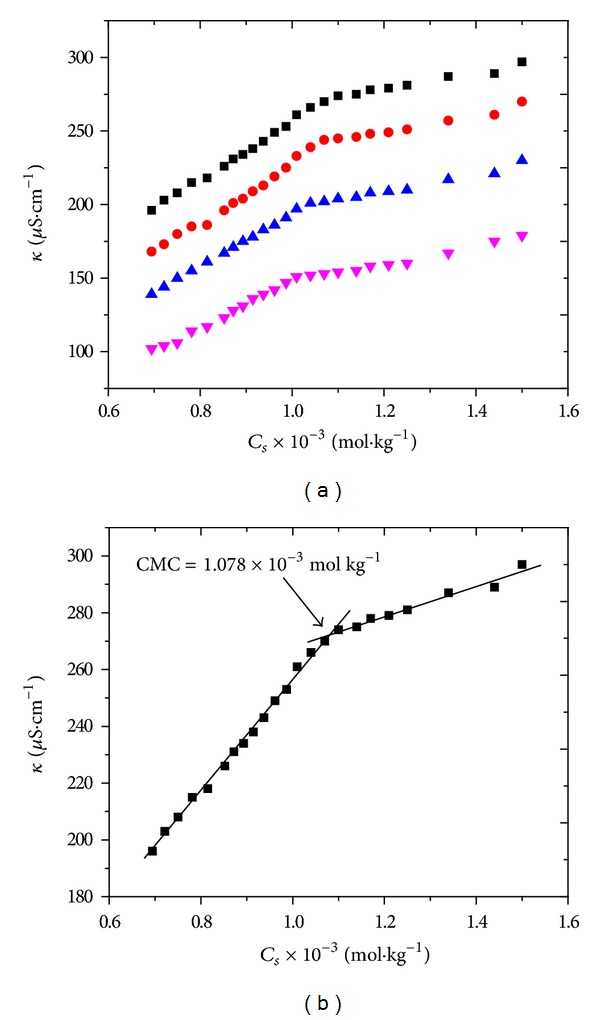
(a) Plots of electrical conductivity versus concentration of CTAB in presence of reactive orange 122 at 298 K (black square), 308 K (red circle), 318 K (blue triangle), and 328 K (purple triangle). (b) Plot of electrical conductivity versus concentration of CTAB in the presence of reactive orange 122 at 298 K.

The electrical conductivity measurement is a reliable and sensitive way to detect CMC and to calculate thermodynamic parameters. In practice, a sharp change in the conductivity of amphiphilic solution is usually observed after reaching CMC. The results from the conductivity-concentration plot (Figure 1) display an evident decrease in the slope, after reaching CMC primarily due to the reasons that (1) micelles are less mobile and (2) concentration of free ions in the solution decreases.

When CTAB molecules dissociate in the premicellar region in water, a dynamic equilibrium is usually established between the undissociated and dissociated molecules as follows;

(9) $$\begin{array}{c} \text{CTAB}⟷{\text{CTA}}^{+}+{\text{B}}^{-} \end{array}$$

In postmicellar region an electrical double layer is often formed around the micelle due to the adsorption of counter ions, where, positive charge is developed at the micelle surface due to unequal distribution of charges between the aqueous and micellar phases. According to Stern's model, the electrical double layer has two parts: (1) a layer of strongly held ions adsorbed onto the micellar surface called Stern layer and (2) a diffused layer of counter ions. In such a case, the electrical potential rapidly drops first within the Stern layer and gradually in diffused layer [14].

The plots of electrical conductivity of CTAB in the presence of reactive orange 122 at various temperatures are shown in Figure 1 while similar plots for reactive red 223 are shown in supplementary Figure S_1_ available online at http://dx.doi.org/10.1155/2014/540975.

Table 1 outlines the data of critical micelle concentrations (CMC), enthalpy of micellization (Δ*H*), free energy of micellization (Δ*G*), entropy of micellization (Δ*S*), and degree of dissociation (*β*) for aqueous solution of CTAB in the presence of reactive orange 122 at different temperatures. The CMC value of pure CTAB is 1.0 mM. Reactive orange 122 causes slight increase in CMC of CTAB due to its structure breaking effect. RO122 has relatively more rigid structure because it has four fused rings; therefore it is difficult to adjust its molecules in micelle; thus its orientation is more likely in outer portion of micelle close to micelle water interface. Such fashion of adsorption of drug molecules increases work of micellization by producing less increase in entropy, thus making micellization less convenient and increasing CMC. RR223 on the other hand has less rigid structure; thus it adjusts more easily in micelle, thus decreasing repulsion between ionic heads of micelle, making micellization easy, and decreasing the CMC value of surfactant [5, 8–10].

From Table 1, the observed continuous decrease in the CMC values with increasing temperature can be explained by the greater degree of hydrophobic dehydration compared to the hydrophilic dehydration. In addition, the changing trends in other thermodynamic parameters such as the large negative value of Δ*G* indicates toward the spontaneous nature of micellization, while, the positive values of Δ*S* and Δ*H* suggest that the micellization was entropy driven process. Literature suggests that the enthalpy change (Δ*H*) during micellization is a combined effect of the changes in enthalpies that arose from hydrophobic interactions, electrostatic interactions, hydration of polar groups, and counter ion binding to the micelles [8–16]. On the other hand, the positive values of Δ*S* are suggested to be due to the transfer of hydrophobic groups of the surfactant from aqueous phase to micelle core. In the immediate vicinity of hydrophobic groups, the strength of hydrogen bonding between water molecules is relatively high. This in turn has a strong effect on the hydrophobic hydration phenomenon, making it different from the usual solvent-solute interaction. The enhanced hydrogen bonding between water molecules in the neighborhood of nonpolar parts leads to the tightening of water structure around hydrophobic groups. Hydrophobic hydration causes internal torsional vibrations of chains to be restricted in the solution. Both of aforementioned factors lead to the decrease in entropy of system. The removal of hydrophobic groups from aqueous media is entropically favorable that causes the disruption of highly organized water structure and removal of mobility constraints on hydrocarbon chain [16–23].

Tables 1 and 2 display various thermodynamic parameters calculated for CTAB in presence of RO122 and RR223, respectively.

### 4.2. Simple Absorption Spectra

**Figure 2:**
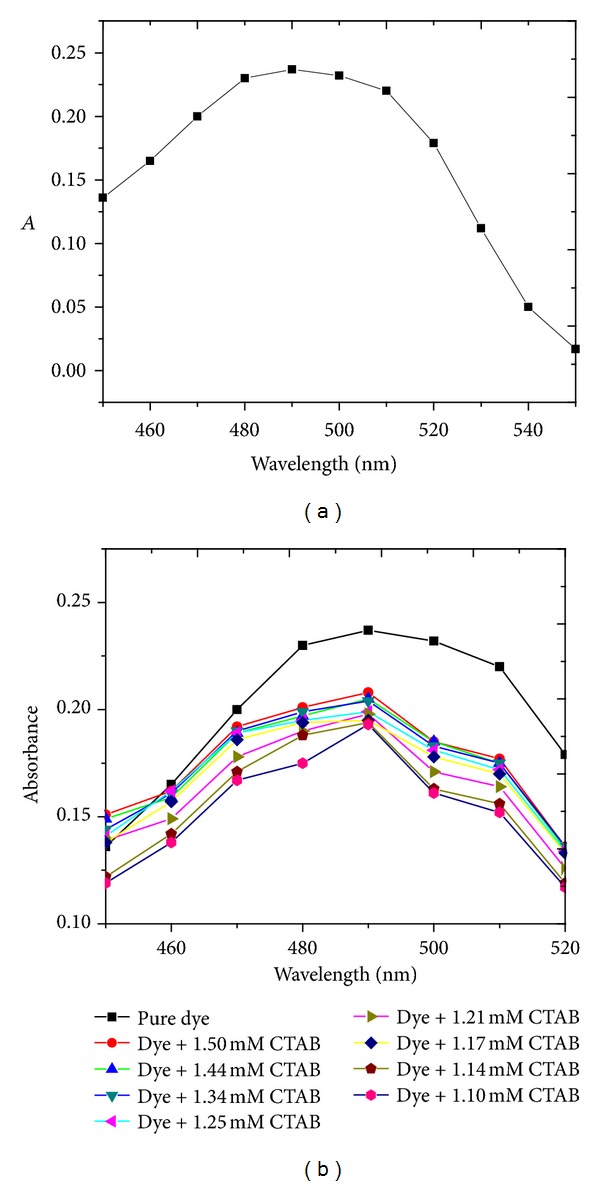
(a) Simple UV/visible absorption spectra of pure reactive orange 122. (b) Simple UV/visible absorption spectra of reactive orange 122 in the presence of different CTAB concentration (mol*·*dm^−3^).

**Figure 3:**
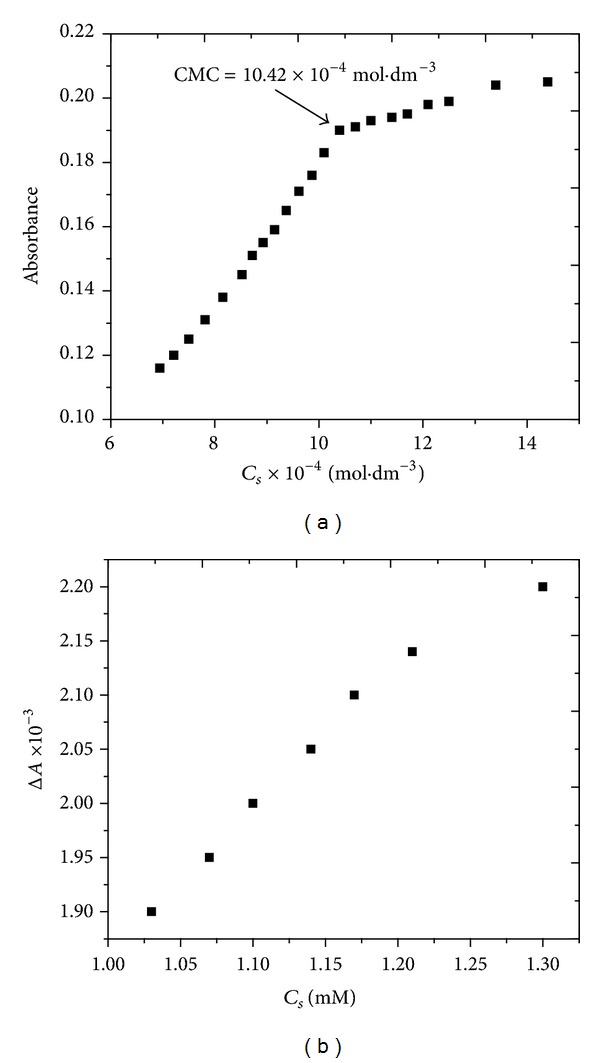
(a) Plot of simple absorbance of reactive orange 122 as a function of CTAB concentration. (b) Plot of differential absorbance of reactive orange 122 as a function of CTAB concentration.

Figure 2 shows UV/visible spectra of reactive orange 122 in the absence (a) and presence of CTAB (b). In both cases, the dye showed an absorption maximum at 490 nm. The effect of surfactant concentration on dye solubilization was clearly observed from the shifts in the absorbance with varying CTAB concentration (Figure 2(b)). This solubilization process can be explained by the host guest relationship between the dye and surfactant molecules, where anionic dye molecules are accommodated within the micelles of cationic CTAB. The increase in absorbance with increasing CTAB concentration (Figure 3(a)) suggests that more dye molecules are getting engaged into the micelle. Once the CMC is reached, no further increase in absorbance occurred, a possible cause of which could be maximum incorporation of dye molecules into the micelle. Formation of new micelles and incorporation of dye molecules into these micelles do not let the absorbance acquire constant value [24–26]. Similar plots of UV/visible spectrum of reactive red 223 with and without CTAB are given in Figure S_2_ (supplementary information).

It has been found that the when the dye molecules interact with the hydrophilic groups of surfactant, it leads to the shift in dimer ↔ monomer equilibrium toward monomer. In such a case, the structural environment of dye molecules changes with CTAB concentration till the CMC is reached, whereas in postmicellar region, no change in the environment takes place [13, 15, 24, 25]. The incorporation of dye molecules into micelles is facilitated because anionic dyes are attracted by cationic CTAB.

There is a continuum of environment from the hydrated micelle surface to the nonpolar core. The solubilizate may be adsorbed onto the surface and orientate itself near the surface (short penetration), or entrapped in the hydrocarbon core (deep penetration). Its polar and/or nonpolar groups interact selectively with the surfactant depending on its substituents. Initially the dye-surfactant complex is formed which is then adsorbed onto the micellar surface and subsequently leads to the reorientation of dye molecules into micelle. The long range electrostatic forces and short range hydrophobic forces act together in the formation of dye surfactant complex. The former bring molecules of dye and surfactant close to each other, while the latter align their hydrophobic parts in parallel fashion:

(10) $$\begin{array}{c} {\text{D}}^{n-}+{\text{S}}^{+}⟷\text{DS} \end{array}$$

With the passage of time, these complexes undergo self-aggregation (as given below) and consequently, increase in the absorbance is witnessed in premicellar region:

(11) $$\begin{array}{c} n\left(\text{DS}\right)⟷{\left(\text{DS}\right)}_{n} \end{array}$$

When the CMC is approached, all dye molecules are compartmentalized in micelles as normal monomers, and at this point the absorbance reaches to its limiting value:

(12) $$\begin{array}{c} {\left(\text{DS}\right)}_{n}+\text{M}⟷\text{DM} \end{array}$$

In (12), DM represents dye monomers that are intercalated into the micelles. Solubilization is a dynamic process, and solubilizate may spend different residence time at different levels between the core and the surface. The imbalance of hydrophilic and hydrophobic forces keeps the solubilizate somehow dynamic in the micelle, thus giving random values of absorbance [4, 14, 26–29]. The hydrophilic-hydrophobic forces influence the orientation of additive molecules at the interface of micelle. In case of polar additives, this balance is prevalent for hydrophilic forces, and thus additive molecules are oriented near the surface of micelle. For nonpolar additives, however, the hydrophobic interactions are favored, and additive molecules are deeply buried inside core of micelle [27, 28].

### 4.3. The Differential Spectroscopy

Differential spectroscopy is a useful tool to calculate the values of partition and binding parameters. A continuous increase in the differential absorption of RO122 with increasing CTAB concentration is indicative of the continuous intercalation of additive molecules within the micelle [7–11, 30]. Similar plots for RR 223 are displayed in Figure S_3_ (supplementary information) for reference. Value of Δ*A* is zero in premicellar region of Figure 3(b) and Figure S_3_(b) because solubilization takes place in postmicellar region [30].

**Figure 4:**
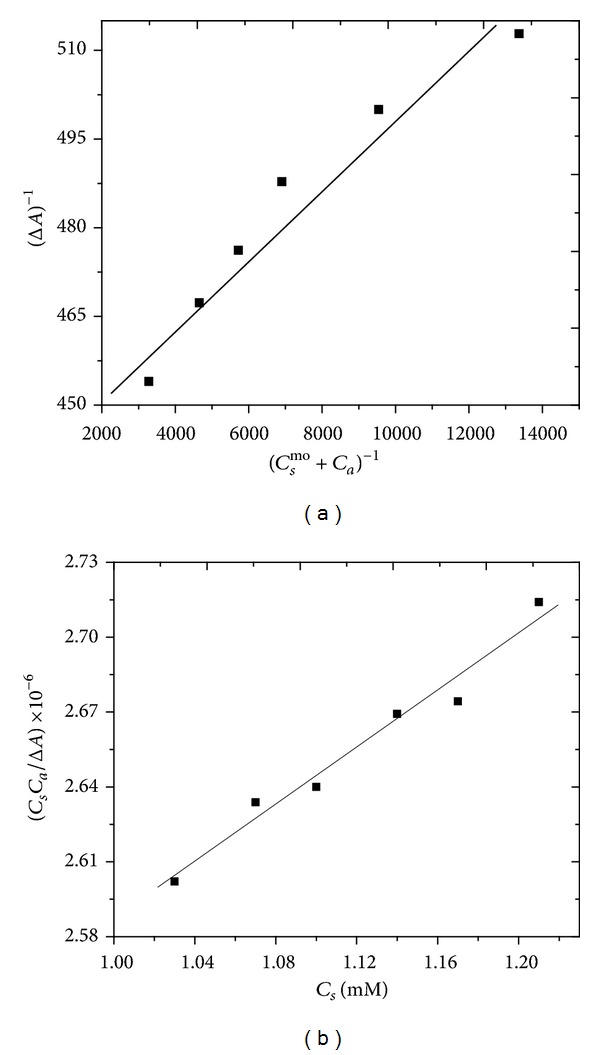
(a) Relationship between (Δ*A*)^−1^ and (*C*_*s*_+*C*_*s*_^*mo*^)^−1^ for the calculation of partition coefficient (*K*_*x*_) for RO122/CTAB system. (b) Plot for calculation of binding constant (*K*_*b*_) for RO122/CTAB system.

The partition coefficient is the ratio of concentration of dye molecules in micelle to that in aqueous solution, whereas, the partitioning of dye molecules between the two phases is governed by partition law. The partition coefficient of solubilizate between micellar and aqueous phase provides information about the extent of solubilization. The value of free energy of partition becomes more negative for more hydrophobic dye, which in turn is an indicator of the ease of penetration of the additives into the micelle. Figures 4(a) and 4(b) display the plots for calculation of partition coefficient and binding constant for RO122, respectively. Similar plots for RR223 are given in Figure S_4_ (supplementary information).

Table 3 displays the values of partition and binding parameters for RO122/CTAB system. The significantly large value of *K*_*x*_  (4.3 × 10^6^) implies large-scale transfer of the dye molecules from the aqueous to micellar media, whereas the large negative value of Δ*G*_*p*_ (−37.84 kJ mol^−1^) is indicative of the spontaneous nature of partitioning. In the same way, the spontaneous nature of dye-surfactant binding process is also evident, as can be observed from the negative value of the free energy of binding (Δ*G*_*b*_ = −14.1 kJ mol^−1^) [3, 8–10, 16, 28, 30–32].

The values of various thermodynamic parameters for RR223/CTAB system are given in Table 4. Again, the values of *K*_*x*_ and *K*_*b*_ were found to be quite high (9.5 × 10^6^ and 600, resp.) because solubilization and binding took place at large scale. It is also suggested that the large negative values of Δ*G*_*p*_ and Δ*G*_*b*_ (−39.8 kJ mol^−1^ and −15.8 kJ mol^−1^, resp.) are due to spontaneous nature of partitioning and binding. Here, the negative values of Δ*G*_*p*_ and Δ*G*_*b*_ are indicative of the stability of system [3, 8–10, 16, 28, 30–32].

**Figure 5:**
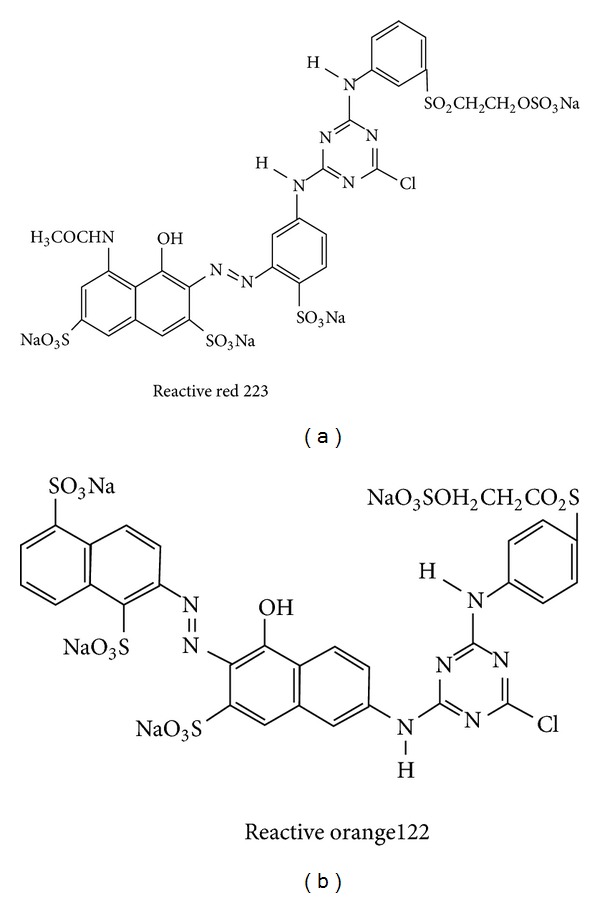


**Figure 6:**
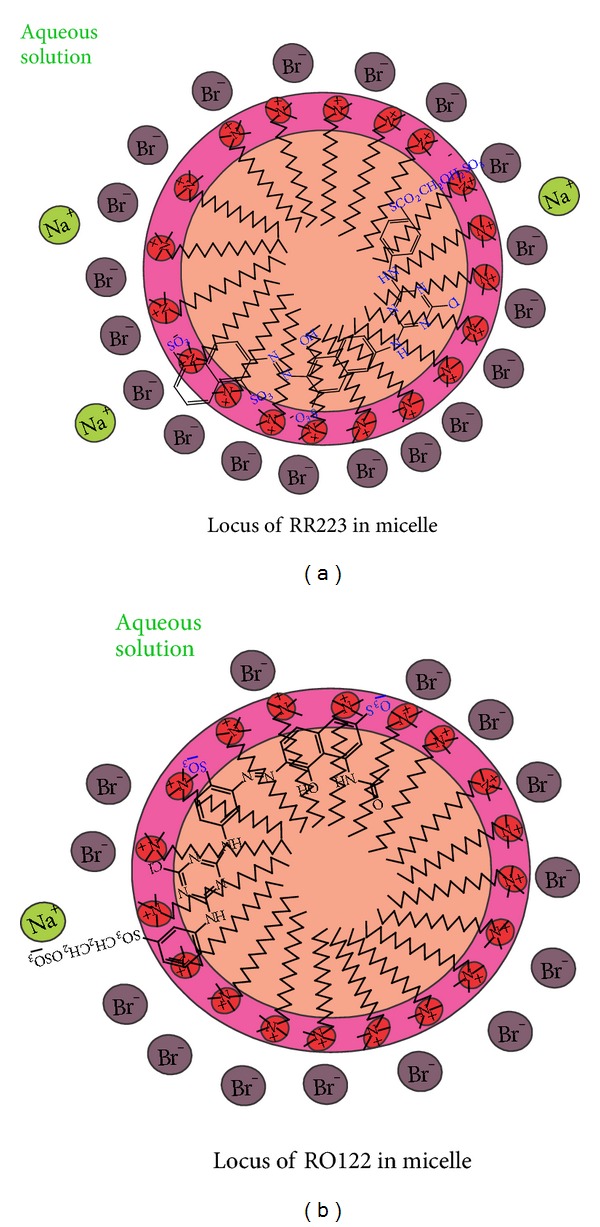


Figures 5(a) and 5(b) show structure of reactive red 223 and reactive orange 122 respectively while Figures 6(a) and 6(b) show proposed locus of dye molecules in micelle. Micelle surface has positive charge while its core is nonpolar. Dye molecules are incorporated in micelle in such a way that their nonpolar parts having aromatic rings will be buried inside nonpolar core of micelle while anionic heads will be attracted by cationic surface of micelle [8–10, 12, 31, 32].

## 5. Conclusions

The present study describes the thermodynamic and spectroscopic investigation of the interactions that occur between the selective anionic dyes (reactive orange 122 and reactive red 223) and the micellar media of CTAB (cationic surfactant). The results from experimental data reveal that the CMC of CTAB in the presence of dyes decreases with increasing temperature due to the preferable dehydration of hydrophobic parts as compared to the hydrophilic ones. The trends of change in various thermodynamic parameters such as Δ*G*, Δ*H*, and Δ*S* suggest that the process of dye solubilization in the micellar media of CTAB is entropy driven and spontaneous. Moreover, the spectroscopic results demonstrate high degree of solubilization and strong host guest relationship between the dyes and surfactant, whereas, the negative values of Δ*G*_*p*_ and Δ*G*_*b*_ indicate spontaneous nature of solubilization and binding.

## Supplementary Material

The of electro conductivity show that RR223 decreases CMC of CTAB. The value of CMC decreases with temperature. The large negative value of ΔG indicates the spontaneous nature of micellization, while, the positive values of ΔS and ΔH suggest that the micellization was entropy driven process. The positive value of ΔH shows that micellization of CTAB in presence of RR223 is endothermic process while the positive values of are due to the transfer of hydrophobic groups of the surfactant from aqueous phase to micelle core.
This solubilization of RR223 by CTAB micelles is due to the host guest relationship between the dye and surfactant molecules, where anionic dye molecules are accommodated within the micelles of cationic CTAB. The increase in absorbance with increasing CTAB concentration suggests that more dye molecules are getting engaged into the micelle. Once the CMC is reached, no further increase in absorbance occurred; a possible cause of which could be maximum incorporation of dye molecules into the micelle.
For RR223/CTAB system the values of partition coefficient, K_x_ and binding constant, K_b_ were found to be quite high (9.5×10^6^ and 600 respectively) because solubilization and binding took place at large scale. It is also suggested that the large negative values of ΔG_p_ and ΔG_b_ (−39.8 kJ mol^−1^ and −15.8 kJ mol^−1^ respectively) are due to spontaneous nature of partitioning and binding.

## Figures and Tables

**Table 1:** The data of critical micelle concentrations (CMC), enthalpy of micellization (Δ*H*), free energy of micellization (Δ*G*), entropy of micellization (Δ*S*), and degree of dissociation (*β*) for aqueous solution of CTAB in the presence of reactive orange 122 at different temperatures.

*T* (K)	CMC (mM)	Δ*H* (kJmol^−1^)	Δ*G* (kJmol^−1^)	Δ*S* (JK^−1^mol^−1^)	*β*
298	1.10	3.56	−46.14	166.75	0.28
308	1.078	3.78	−47.54	166.60	0.29
318	1.04	3.91	−47.77	162.50	0.34
328	1.01	4.08	−48.50	160.32	0.37

**Table 2:** The data of critical micelle concentrations (CMC), enthalpy of micellization (Δ*H*), free energy of micellization (Δ*G*), entropy of micellization (Δ*S*), and degree of dissociation (*β*) for aqueous solution of CTAB in the presence of reactive red 223 at different temperatures.

*T* (K)	CMC (mM)	Δ*H* (kJmol^−1^)	Δ*G* (kJmol^−1^)	Δ*S* (JK^−1^mol^−1^)	*β*
298	0.94	5.46	−15.26	69.54	0.493
308	0.87	5.72	−15.75	69.72	0.515
318	0.82	6.06	−16.39	70.56	0.532
328	0.815	6.36	−16.69	70.27	0.553

**Table 3:** The data of partition coefficient (*K*_*x*_), free energy of partition (Δ*G*_*x*_), binding constant (*K*_*b*_), and the free energy of binding (Δ*G*_*b*_) for reactive orange 122/CTAB system.

10^−6^ *K*_*x*_	Δ*G*_*x*_/(kJmol^−1^)	*K*_*b*_/(dm^3^ mol^−1^)	Δ*G*_*b*_/(kJ mol^−1^)
4.30	−37.84	300	−14.1

**Table 4:** The data of partition coefficient (*K*_*x*_), free energy of partition (Δ*G*_*x*_), binding constant (*K*_*b*_), and free energy of binding (Δ*G*_*b*_) for reactive red 223/CTAB system.

10^−6^ *K*_*x*_	Δ*G*_*x*_/(kJ mol^−1^)	*K*_*b*_/(dm^3^ mol^−1^)	Δ*G*_*b*_/(kJ mol^−1^)
9.5	−39.8	600	−15.8

## References

[1] Gul F, Khan AM, Shah SS, Nazar MF (2010). Spectroscopic study of alizarin red s binding with cetyltrimethylammonium bromide at low concentrations. *Coloration Technology*.

[2] Tehrani-Bagha AR, Holmberg K (2013). Solubilization of hydrophobic dyes in surfactant solutions. *Materials*.

[3] Shah SS, Khan MS, Ullah H, Awan MA (1997). Solubilization of amphiphilic hemicyanine dyes by a cationic surfactant, cetyltrimethylammonium bromide. *Journal of Colloid and Interface Science*.

[4] Dezhampanah H, Firouzi R (2012). Thermodynamic investigation of the interaction between anionic dye and cationic surfactant in aqueous solution. *International Journal of Research in Physical Chemistry*.

[5] Naseem B, Sabri A, Hasan A, Shah SS (2004). Interaction of flavonoids within organized molecular assemblies of anionic surfactant. *Colloids and Surfaces B: Biointerfaces*.

[6] Needles HL (1986). *Textil Fibres, Dyes, Finishes, and Processes: A Concise Guide*.

[7] Tappe H, Helmling W, Mischke P (2000). Reactive dyes. *Ullmann's Encyclopedia of Industrial Chemistry*.

[8] Usman M, Siddiq M (2013). Surface and micellar properties of chloroquine diphosphate and its interactions with surfactants and human serum albumin. *Journal of Chemical Thermodynamics*.

[9] Usman M, Siddiq M (2013). Probing the micellar properties of Quinacrine 2HCl and its binding with surfactants and Human Serum Albumin. *Spectrochimica Acta A*.

[10] Usman M, Rashid MA, Mansha A, Siddiq M (2013). Thermodynamic solution properties of pefloxacin mesylate and its interactions with organized assemblies of anionic surfactant, sodium dodecyl sulphate. *Thermochimica Acta*.

[11] Shah A, Khan AM, Usman M, Qureshi R, Siddiq M, Shah SS (2009). Thermodynamic characterization of dexamethasone sodium phosphate and its complex with dna as studied by conductometric and spectroscopic techniques. *Journal of the Chilean Chemical Society*.

[12] Kawamura H, Manabe M, Miyamoto Y, Fujita Y, Tokunaga S (1989). Partition coefficients of homologous *ω*-phenylalkanols between water and sodium dodecyl sulfate micelles. *Journal of Physical Chemistry*.

[13] Mehta SK, Chaudhary S, Bhasin KK, Kumar R, Aratono M (2007). Conductometric and spectroscopic studies of sodium dodecyl sulfate in aqueous media in the presence of organic chalcogen. *Colloids and Surfaces A: Physicochemical and Engineering Aspects*.

[14] Rosen MJ (1973). *Surfactants and Interfacial Phenomenon*.

[15] Mehta SK, Bhasin KK, Kumar A, Dham S (2006). Micellar behavior of dodecyldimethylethyl ammonium bromide and dodecyltrimethylammonium chloride in aqueous media in the presence of diclofenac sodium. *Colloids and Surfaces A: Physicochemical and Engineering Aspects*.

[16] Naseem B (2006).

[17] Taboada P, Martínez-Landeira P, Ruso JM, García M, Mosquera V (2002). Aggregation energies of some amphiphilic antidepressant drugs. *Colloids and Surfaces A*.

[18] Cheema MA, Taboada P, Barbosa S, Castro E, Siddiq M, Mosquera V (2006). A thermodynamic study of the amphiphilic phenothiazine drug thioridazine hydrochloride in water/ethanol solvent. *Chemical Physics*.

[19] Cheema MA, Taboada P, Barbosa S, Siddiq M, Mosquera V (2006). Effect of molecular structure on the hydration of structurally related antidepressant drugs. *Molecular Physics*.

[20] Cheema MA, Taboada P, Barbosa S, Castro E, Siddiq M, Mosquera V (2007). Thermodynamic study of warfarin sodium salt: surface tension, conductivity and density measurements. *Journal of Chemical & Engineering Data*.

[21] Cheema MA, Siddiq M, Barbosa S, Taboada P, Mosquera V (2008). Surface and bulk properties of two amphiphilic phenothiazine drugs in different aqueous media. *Journal of Chemical and Engineering Data*.

[22] Khan AM, Shah SS (2008). Determination of critical micelle concentration (Cmc) of sodium dodecyl sulfate (SDS) and the effect of low concentration of pyrene on its Cmc using ORIGIN software. *Journal of the Chemical Society of Pakistan*.

[23] Attwood D, Florence AT (1985). *Surfactant Systems*.

[24] Łuczak J, Jungnickel C, Joskowska M, Thöming J, Hupka J (2009). Thermodynamics of micellization of imidazolium ionic liquids in aqueous solutions. *Journal of Colloid and Interface Science*.

[25] Mishra A, Behera PK, Behera RK, Mishra BK, Behera GB (1998). Interaction of N-alkyl styryl pyridinium dyes with TX-100 in aqueous medium: role of the alkyl chain during solubilisation. *Journal of Photochemistry and Photobiology A: Chemistry*.

[26] Schick MJ, Fowkes FM (1957). Foam stabilizing additives for synthetic detergents. Interaction of additives and detergents in mixed micelles. *Journal of Physical Chemistry*.

[27] Sarkar M, Poddar S (2000). Studies on the interaction of surfactants with cationic dye by absorption spectroscopy. *Journal of Colloid and Interface Science*.

[28] Khan AM, Shah SS (2008). A UV-visible study of partitioning of pyrene in an anionic surfactant sodium dodecyl sulfate. *Journal of Dispersion Science and Technology*.

[29] Akbaş H, Taner T (2009). Spectroscopic studies of interactions between C.I. Reactive Orange 16 with alkyltrimethylammonium bromide surfactants. *Spectrochimica Acta A*.

[30] Naeem K, Shah SS, Shah SWH, Laghari GM (2000). Solubilization of cationic hemicyanine dyes in anionic surfactant micelles: a partitioning study. *Monatshefte fur Chemie*.

[31] Shah SS, Laghari GM, Naeem K, Shah SWH (1998). Partition coefficient of amphiphilic hemicyanine dyes between the aqueous and the micellar phase of sodium dodecyl sulfate by differential absorbance spectroscopy. *Colloids and Surfaces A: Physicochemical and Engineering Aspects*.

[32] Shah SS, Laghari GM, Naeem K, Shah SWH (2000). Differential absorbance measurements of amphiphilic hemicyanine dyes, solubilization study in anionic surfactant. *Colloids and Surfaces A*.

