# Supplementary material for: Thermodynamic and Spectroscopic Investigation of Interactions between Reactive Red 223 and Reactive Orange 122 Anionic Dyes and Cetyltrimethyl Ammonium Bromide (CTAB) Cationic Surfactant in Aqueous Solution
Source: ScientificWorldJournal. 2014 Aug 28;2014:540975. doi: 10.1155/2014/540975 (PMC4163323; doi:10.1155/2014/540975)
Supplement: Supplementary file 1 — The of electro conductivity show that RR223 decreases CMC of CTAB. The value of CMC decreases with temperature. The large negative value of ΔG indicates the spontaneous nature of micellization, while, the positive values of ΔS and ΔH suggest that the micellization was entropy driven process. The positive value of ΔH shows that micellization of CTAB in presence of RR223 is endothermic process while the positive values of are due to the transfer of hydrophobic groups of the surfactant from aqueous phase to micelle core. This solubilization of RR223 by CTAB micelles is due to the host guest relationship between the dye and surfactant molecules, where anionic dye molecules are accommodated within the micelles of cationic CTAB. The increase in absorbance with increasing CTAB concentration suggests that more dye molecules are getting engaged into the micelle. Once the CMC is reached, no further increase in absorbance occurred; a possible cause of which could be maximum incorporation of dye molecules into the micelle. For RR223/CTAB system the values of partition coefficient, Kx and binding constant, Kb were found to be quite high (9.5×10^6^ and 600 respectively) because solubilization and binding took place at large scale. It is also suggested that the large negative values of ΔGp and ΔGb (−39.8 kJ mol−1 and −15.8 kJ mol−1 respectively) are due to spontaneous nature of partitioning and binding. [file 540975.f1.docx]

Figure S_1_.

Figure S_2_

Figure S_3_

Figure S_4_

**Captions of Supplementary Figures**

Figure S_1_. Plots of electrical conductivity versus concentration of CTAB in presence of Reactive Red 223 at 298K ($∎$), 308K ($\circ$), 318K ($\Delta)$ and 328K ($\nabla).$ 3(b). Plot of electrical conductivity versus concentration of CTAB in presence of Reactive Red 223 at 298K

Figure S_2_. (a) Simple UV/Visible absorption spectra of pure Reactive red 223 (b) Simple UV/Visible absorption spectra of Reactive red 223 in the presence of different CTAB concentration (mol.dm^-3^)

Figure S_3_. (a) Plot of simple absorbance of Reactive red 223 as a function of CTAB concentration. (b) Plot of differential absorbance of Reactive red 223 as a function of CTAB concentration.

Figure S_4_. Relationship between (ΔA)^-1^ and (C_s_ +C_s_^mo^)^-1^ for the calculation of partition coefficient (K_x_) for RR223/CTAB system. (b) Plot for calculation of binding constant (K_b_) for RR223/CTAB system.
